# Successful pregnancy following conservative management of low-grade endometrial stromal sarcoma: A case report

**DOI:** 10.3892/ol.2014.1858

**Published:** 2014-02-07

**Authors:** RUIYING DONG, YINGXIN PANG, HONGLUAN MAO, NING YANG, PEISHU LIU

**Affiliations:** Department of Obstetrics and Gynecology, Qilu Hospital of Shandong University, Jinan, Shandong 250012, P.R. China

**Keywords:** endometrial stromal sarcoma, endocrine therapy, fertility-preserving surgery, pregnancy

## Abstract

It is uncommon that fertility is preserved in young nulliparous females with low-grade endometrial stromal sarcoma (ESS). Therefore, successful pregnancy following such conservative management has been rarely reported in previous literature. A 25-year-old female (gravida, 0; para, 0) underwent hysteroscopic surgery and was pathologically diagnosed with an endometrial stromal nodule. The patient underwent fertility-preserving local resection and uterine reconstruction, with a final pathological diagnosis of low-grade ESS. Endocrine therapy was then administered. Conservative management resulted in the complete remission of low-grade ESS. The patient naturally conceived and successfully delivered a healthy baby at 42 weeks’ gestation by cesarean section, ~30 months following diagnosis with low-grade ESS. In conclusion, conservative management, including fertility-preserving local mass resection and endocrine therapy, can be effective for low-grade ESS and may yield a favorable outcome for young nulliparous females desiring fertility preservation.

## Introduction

Endometrial stromal sarcoma (ESS) is a rare malignancy and accounts for <1% of all uterine malignancies and ~15% of all uterine sarcomas (1). In general, ESS occurs in perimenopausal and postmenopausal females (2,3), and <25% of ESS patients are premenopausal (4). The mainstay of treatment for ESS is total hysterectomy and bilateral salpingo-oophorectomy, and adjuvant treatments include chemotherapy, radiation and endocrine therapy. ESS is classified into two subtypes, high- and low-grade ESS, mainly on the basis of pathological mitotic index and cytologic atypia (4). Considering that low-grade ESS is associated with a markedly more favorable prognosis compared with other uterine sarcomas, fertility-preserving treatment in young nulliparous females is feasible. However, due to the rarity of the disease, experience of conservative management in low-grade ESS is limited (5).

The current report presents a case of low-grade ESS which was initially misdiagnosed as submucous myoma according to the ultrasonic examination. The patient was diagnosed with an endometrial stromal nodule following primary hysteroscopic surgery and was eventually diagnosed with low-grade ESS at the subsequent laparotomy. A desired outcome was achieved; the patient successfully conceived and delivered a healthy baby following the second local excision and endocrine therapy.

## Case report

A 25-year-old female was admitted to the Qilu Hospital of Shandong University (Jinan, China) complaining of menorrhagia and shortened menstrual cycles of three months and acute lower abdominal pain lasting for 10 h. The patient had a surgical history of right breast fibroadenoma removal at 23 years of age. The patient’s medical history was unremarkable. A previous B ultrasound examination demonstrated a hypoechoic lesion (6.1×5.2×5.9 cm) with a clear borderline on the left posterior wall of the uterus. Physical examination of the external genitalia, vagina and cervix showed no abnormalities. Pelvic examination revealed a regularly enlarged uterus, the size of 70 days pregnancy. No adnexal masses were palpated. Primary diagnosis was determined as submucous myoma and a myomectomy was performed by hysteroscopy. During surgery, a submucous mass that resembled type II myoma was found in the left posterior wall of the patient’s uterus, with a hemorrhagic and necrotic surface. Due to the incomplete resection, as a section of the mass was in the wall, subsequent surgery was suggested. One week later, histological results of the formalin-fixed resected mass confirmed a submucous endometrial stromal nodule, immunohistochemically positive for CD10. The patient recovered satisfactorily and was discharged from hospital six days later.

**Figure 1:**
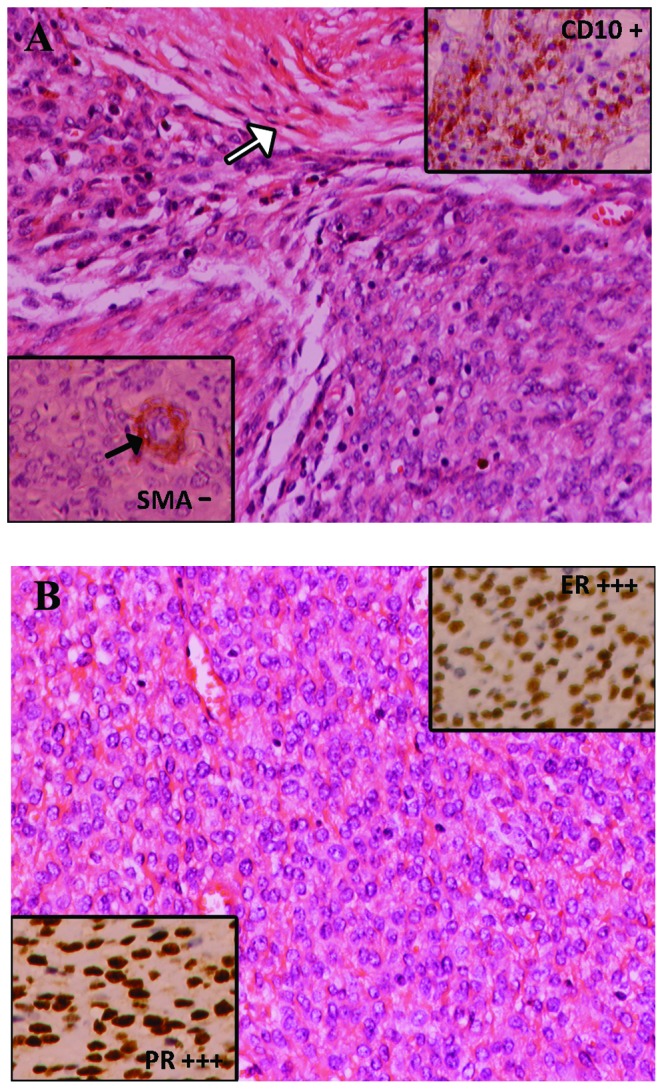
Microscopic and immunohistochemical features of the resected tissue (magnification, ×20). (A) Invasion of the myometrium by endometrial stromal sarcoma. Tumor cells showed CD10 positivity and SMA negativity. White arrow indicates the area of stromal sarcoma cells infiltrating the myometrium. Black arrow indicates that the blood vessel wall was markedly positive for SMA, as a positive control, while tumor cells revealed SMA negativity. (B) Strong positivity for estrogen receptor and progesterone receptor in the nucleus of stromal sarcoma cells (brown). SMA, smooth muscle actin.

Three months later, the patient returned to the hospital for a laparotomy in order to resect the residual lesions. The surgery was performed on May 31, 2010. During surgical exploration, a convex mass with a diameter of 5 cm was detected on the posterior wall of the enlarged uterus and the decision was made to perform resection. The mass, which was detected to be trans-endometrial and well-circumscribed from the surrounding myometrium, was completely resected. Histological examination of frozen sections of the resected mass during the surgery suggested low-grade ESS. Thus, radical hysterectomy was proposed to the patient’s family, but the family rejected and expressed a desire for fertility preservation since the patient was nulliparous. As there were no signs of tumor infiltration and metastasis, the surgical procedure was changed to preserve and reconstruct the uterus, which was successfully accomplished. Final pathological examination of the formalin-fixed resected tissue (4.5×3.3 cm) confirmed the diagnosis of low-grade ESS. Various levels of staining intensity are indicated by −, +, ++ and +++; negative, weak, moderate and strong, respectively Immunohistochemistry showed CD10(+), desmin(+), smooth muscle actin (SMA)(−) and 30% Ki67(+). Further detection of estrogen receptor (ER), progesterone receptor (PR) and p53 by immunohistochemistry showed ER(+++), PR(+++) and p53(−) (Fig. 1).

Chemotherapy and endocrine therapy were suggested as adjuvant treatments, but the patient declined chemotherapy considering its side effects and the patient’s desire for fertility. Medroxyprogesterone (250 mg) was administered daily for one year to inhibit tumor recurrence. No abnormal abdominal or pelvic observations were identified.

Following six months of medroxyprogesterone withdrawal, the patient conceived naturally. Pregnancy was uncomplicated and the patient delivered a healthy baby of 3,300 g at 42 weeks’ gestation by cesarean section on November 22, 2012. On January 9, 2013, the patient returned to the gynecological clinic of the hospital for a general check-up and no signs of tumor recurrence were identified. Written informed consent was obtained.

## Discussion

ESS is a rare malignant disease with an overall five-year survival rate of ~30% (6). ESS accounts for only 15% of uterine sarcomas. Due to its infrequency, ESS is commonly mistaken for leiomyomas (7). Common clinical signs of ESS include abnormal uterine bleeding, menorrhagia and pelvic pain, which were all present in the current case report. ESS is usually classified into low- and high-grades and the latter generally involves perimenopausal and postmenopausal females who exhibit blood metastasis at an early stage and relatively poor prognosis. Compared with high-grade ESS in which mitotic figures are common by microscopic examination, low-grade ESS has a tendency of local recurrence with an indolent growth and its mitotic figures are usually less than five per 10 high-power fields. The median time of recurrence is 65 months for patients with low-grade ESS at stage I (8).

The mainstay treatment for ESS is total abdominal hysterectomy and bilateral salpingo-oophorectomy. Due to the rarity of ESS in young nulliparous females, the occasional cases of ESS in young patients are usually identified in histological examinations of resected presumed leiomyomas of the uterus (3,9). The current report presents a case of the local resection of a low-grade ESS, in which fertility was preserved for the young nulliparous female. Histopathologically, ESS is frequently misdiagnosed with myoma. CD10 and SMA have been used as immunohistochemical markers for distinguishing between ESS and uterine smooth muscle tumors (10,11). In the current report, it was via the results of immunohistochemical staining (CD10-positive and SMA-negative) that the diagnosis of ESS was determined. Adjuvant treatments of low-grade ESS include chemotherapy, radiation and endocrine therapy, among which endocrine therapy, particularly progestin therapy, is considered the most effective for curing and preventing local recurrence. Endocrine therapy is recommended as a routine adjuvant management for primary and recurrent ESS (12). Considering the high expression levels of ER and PR revealed in the current case report by immunohistological examination, the patient was administered medroxyprogesterone for one year following fertility-preserving surgery. The therapeutic efficacy of medroxyprogesterone was satisfactory. Following six months of drug withdrawal, the patient successfully conceived and delivered a healthy baby at 42 weeks’ gestation.

Previous relevant literature concerning ESS were extensively reviewed. Indeed, fertility-preserving surgery in young nulliparous females had been proposed in several cases (2–5) and successful pregnancy following such management had been reported, shown in detail in Table I (2,3). For patients who present with a confined uterus mass and no signs of tumor metastasis or infiltration, it is, in theory, feasible to preserve the patient’s uterus. However, Koskas *et al* previously reported a case of a 34-year-old female with a diagnosis of low-grade ESS who underwent a severe peritoneal recurrence following the successful delivery of a healthy baby (13). Li *et al* also identified that surgery sparing ovarian function increased the risk of recurrence of ESS compared with those without the preservation of ovarian function (14). In addition, Amant *et al* (15) and Picker *et al* (16) each reported a case of ESS during pregnancy. Although aggressive treatments were performed in the two patients, the patients succumbed to their diseases six days and two years following diagnosis, respectively. A possible explanation was that high levels of circulating estrogen enhanced the progression of the tumor. A previous study also reported that the treatment outcome has a significant association with stage, histological subtype, tumor size and positivity from cytologic biopsy (17). Therefore, conservative management must be performed with caution in young nulliparous patients with ESS. The risk of preserving the uterus must be sufficiently evaluated prior to fertility-preserving surgery and consent of the patient must be obtained preoperatively. Local resection of the uterus mass is inevitable and adjuvant treatment, particularly endocrine treatment, also appears to be necessary as illustrated by current evidence. Thereafter, strict follow-up is required to monitor recurrence.

Although ESS is a rare uterine malignancy with poor prognosis, in selected cases with a local uterus mass and no signs of metastasis and infiltration, conservative management, including local surgical resection and adjuvant treatment, may be performed to preserve fertility. The present case report suggests that fertility preservation by local resection and uterine reconstruction may be a viable option for young females with low-grade ESS.

## Figures and Tables

**Table I:** Systematic review of ESS followed by pregnancy.

Authors (reference)	Age, years	Clinical manifestation/initial symptoms	Surgery	Pathology	Adjuvant therapy	Time to pregnancy	Recurrence	Treatment following recurrence
Delaney *et al* (2)	16	Menorrhagia and abdominal distension	Local resection and uterine construction	Low-grade ESS	Endocrine therapy (megestrol acetate)	8 years	No	NA
Yan *et al* (3)	25	Menorrhagia	Local resection and uterine construction	High-grade ESS	Chemotherapy (etoposide and cisplatin)	40 months	No	NA
Koskas *et al* (13)	34	Infertility	Resection by hysteroscopy	Low-grade ESS	No	6 months	Severe peritoneal resection	Endocrine therapy (non-steroidal aromatase inhibitors

ESS, endometrial stromal sarcoma; NA, not applicable.
